# The Life History and Shelter Building Behavior of *Vettius Coryna Coryna* Hewitson, 1866 in Eastern Ecuador (Lepidoptera: Hesperiidae: Hesperiinae)

**DOI:** 10.1673/031.009.3201

**Published:** 2009-06-02

**Authors:** Harold F. Greeney, Andrew D. Warren

**Affiliations:** ^1^ Yanayacu Biological Station & Center for Creative Studies, Cosanga, Ecuador c/o 721 Foch y Amazonas, Quito, Ecuador; ^2^ McGuire Center for Lepidoptera and Biodiversity, Florida Museum of Natural History, University of Florida, P.O. Box 112710, Gainesville, Florida 32611; ^2^ Museo de Zoología, Facultad de Ciencias, Universidad Nacional Autónoma de México, Apdo. Postal 70-399, México, D.F. 04510 México

**Keywords:** Andes, larva, larval shelter, pupa, skipper

## Abstract

We describe all life-stages of *Vettius coryna coryna* Hewitson, 1866 in eastern Ecuador. The details of larval shelter structure and associated shelter building behavior are described and illustrated, as observed on two grass species (Poaceae). We provide brief observations on *V. coryna* adult behavior and a review of known life history information for other species of *Vettius* Godman, 1901.

## Introduction

The genus *Vettius* Godman, 1901 contains 22 small to medium-sized skipper species, confined to the Neotropics (Evans 1955, Mielke 2004, 2005). The wings of most are rather showy, usually with white spots or patches on a dark background, as well as highlights of yellow, orange, or in the *Vettius coryna* (Hewitson, 1866) species group, metallic silver (Figure 2b). The *V. coryna* group (Evans 1955) includes four taxa of uncertain taxonomic status: *V. argentus* H. A. Freeman, 1969 described from Chiapas, Mexico, *V. conkaEvans*, 1955 described from Guatemala, *V. catargyra* (C. Felder & R. Felder, 1867) described from Venezuela, and *V. coryna* described from “Amazons.” These taxa differ primarily in the number and size of the white forewing spots, especially those located within the discal cell. In the most recent treatment (Mielke 2004, 2005), *coryna, catagryra* and *conka* are treated as subspecies of *V. coryna* following Evans (1955), while *argentus* is given full species status. Nevertheless, the taxonomy of the *Vettius coryna* group requires careful study to determine the actual relationships of the four included taxa.

*Vettius coryna coryna* (Figure 2b), distributed throughout the Andes from Venezuela south to Bolivia (Bebee 1951; Evans 1955), is usually found at elevations above 1500 m and commonly occurs up to 2400 m in Peru (Lamas 2003:33). It is quite common and is frequently collected, presumably because of its attractive, mostly silvered ventral surface. The taxon has been illustrated in color in several widely available identification guides (Smart 1984; Lewis 1987; Piñas and Manzano 1997; Lamas 2003). However, the results presented here are the first published notes on the immature stages of *V. c. coryna*. We hope our study will contribute to a better understanding of systematics within the *V. coryna* group, as well as to relationships between the *V. coryna* group and other members of *Vettius*.

## Materials and Methods

We made all collections and observations of adults and immatures of *V. c. coryna* in eastern Ecuador, at the Yanayacu Biological Station and Center for Creative Studies (YBS; 00°35.95′S, 77°53.40′W), located at an elevation of 2150 m in the Quijos Valley, Napo Province, on the eastern slope of the Andes Mountains. The study site is located approximately five kilometers west of the town of Cosanga and is adjacent to the Cabañas San Isidro preserve of over 2000 hectares of primary cloud forest, bordered by cattle pasture and other disturbed habitats (see Valencia 1995; Greeney et al. 2006).

On 23 November, 1999, HFG visited disturbed forest adjacent to a large cattle pasture and there collected seven early instars, two fourth instars and one fifth (final) instar of *V. c. coryna* on two grass species (Poaceae): *Pennisetum tristachyon* (Kunth) Sprang., and a species of *Paspalum* L., either *P. peniculatum* L. or *P. jurgensii* Hack. We transported larvae in plastic bags to the YBS and reared them in separate, small, plastic containers. We added fresh foodplant daily. Subsequently, we discovered numerous larvae in similar situations at the study site, and treated them as described above. We observed larvae of all stadia in the field as well as in the lab, but made descriptions of shelter building behavior exclusively in the field prior to collection. Terminology for shelter types follows Greeney and Jones (2003). We observed more than 200 larvae, and reared a total of 33 larvae to adult eclosion. Voucher material is deposited in the collections of the authors, as well as at the McGuire Center for Lepidoptera and Biodiversity, Florida Museum of Natural History, University of Florida (Gainesville).

## Results

### Egg (Diameter = 0.9 mm; n = 1)

Dome-shaped, white to pale yellow, appearing smooth but with minute web-like sculpturing forming pentagons and hexagons that cover nearly the entire surface; area around micropyle smooth.

### Larval behavior

Larvae were encountered during all months of the year; all instars constructed and rested in leaf shelters. All instars forcibly ejected frass from the anus, directing it away from the shelter; no frass was encountered inside larval shelters.

### First instar (Figure 1 c; n = 20+)

**Figure 1.**
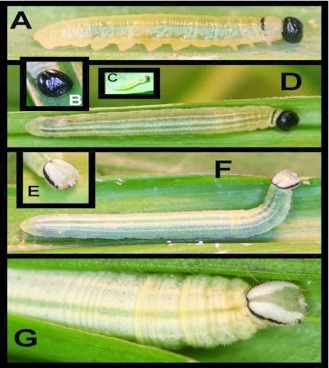
Larvae of *Vettius coryna coryna* at YBS (Napo Province, Ecuador, 2100 m). a) Premolt 2nd instar; b) 3rd instar head; c) 1st instar; d) 4th instar; e) head of 5th instar; f-g) 5th instar.

Head appearing smooth, but with fine reticulations and a sparse covering of short, pale setae visible under magnification, shape nearly round to slightly triangular, epicranial suture not prominent, color dark brown to black; body varying from pale, translucent yellow-white to pale emerald-green, color dependent on gut contents at time of observation; overall shape tubular, abdominal segments slightly laterally produced laterally (Figure 1c); pronotum narrow, extending to edge of dorsal area, similar in color to head.

### Second instar (Figure 1a; n = 20+; body length = to 6 mm)

Extremely similar to first instar, except body now appearing nearly parallel-sided in dorsal view.

### Third instar (Figure 1b; n = 30+; development time = 10–12 days; body length = to 11 mm)

Similar to second instar, except near end of 3rd instar body develops two subdorsal, longitudinal white lines, extending from T2 to A8, two faint, thin dorsolateral white lines extend from T3 to A7; all markings faint.

### Fourth instar (Figure 1d; n = 42; development time = 11–14 days; body length = to 19 mm)

Head appearing smooth, but with fine reticulations when viewed under magnification (as described for earlier instars), roundly triangular with a slight epicranial suture, usually entirely dark brown to black, but in some individuals with a pair of pale, inverted, elongate, triangular spots at apex on either side of suture, and another pair of smaller triangular spots just posterior to stemmata; body elongate, parallel-sided, constricted slightly at T1 and T2, tubular when stretched out or feeding, but widest around A3 and slightly hunch-backed in appearance when resting; ground color emerald-green to pale yellow with four narrow, pale, powdery-white, longitudinal stripes, the dorsal pair slightly wider, extending from T2 to A10, two fainter stripes running sub-dorsally from T3 to A8; males with a pair of blue-green or yellow-green, kidney to oval-shaped testes visible through cuticle on either side of midline around A6–A7; pronotum thin, dark brown to black, extending to ventral edge of subdorsal area; a pale purple-red ventral prothoracic “neck” gland present; when probed, larvae rear back on prolegs and evert prothoracic gland with head tipped back.

### Fifth instar (Figures 1e-1g; n = 41; 21–23 days; to 28 mm)

Head sub-triangular; epicranial suture translucent greenwhite with black bands running laterally from ocular area and meeting dorsally, two broader, white bands anterior to black bands that do not meet dorsally; clypeus bright white to yellow-white, mandibles black; body similar to fourth instar except lacking a strongly scleritized pronotum, markings bolder though becoming less visible as an overall dusting of white to yellow-white develops later in instar; skin folds (especially inter-segmental ones) appearing as white to yellow-white bands; as larva develops and nears pupation, a thick coating of white waxy flocculence appears in four patches on ventral surface of A7 and A8; eversible prothoracic gland present (see description of fourth instar); anal comb lightly sclerotized, roughly scallop-shaped, bearing 19 short spines; a single individual molted into a sixth instar, which was similar in appearance to 5^th^ instar.

### Pupa (Figures 2a, 2c-2d; n = 32; 25–28 days; 27–32 mm)

Elongate, roughly cylindrical, tapering toward cremaster; a prominent, flattened, triangular, anteriorly-directed horn arising between eyes; abdomen and wing pads with a light dusting of white waxy flocculence [as in late 5^th^ instars (above)]; proboscis sheath detached beyond wing pads and extending to base of cremaster; ground color clear lime-green, changing to white or yellow-white several days after pupation; a pair of thin stripes dorsally, extending from prothorax to base of cremaster (which is clear white); two similar sub-dorsal stripes, extending from Al to A8 (where they become faint); some specimens with faint white dashes sub-dorsally on pronotum behind eyes; 3–4 days before adult eclosion, eyes darken to brown and wing pads become bright orange; 1 day before eclosion, thorax and head darken to black, abdomen develops a dark mid-dorsal stripe, and adult wing pattern becomes visible through cuticle.

**Figure 2.**
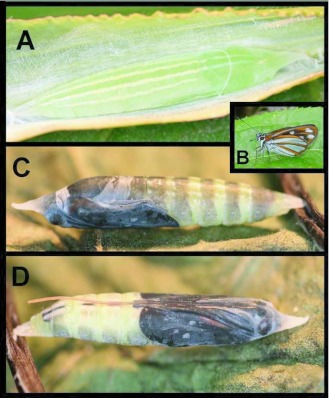
Pupa and adult of *Vettius coryna coryna* at YBS. a) Freshly formed pupa; b) adult; c-d) mature pupa with pharate adult visible through cuticle.

### Larval shelters (Figures 3–4)

**Figure 3.**
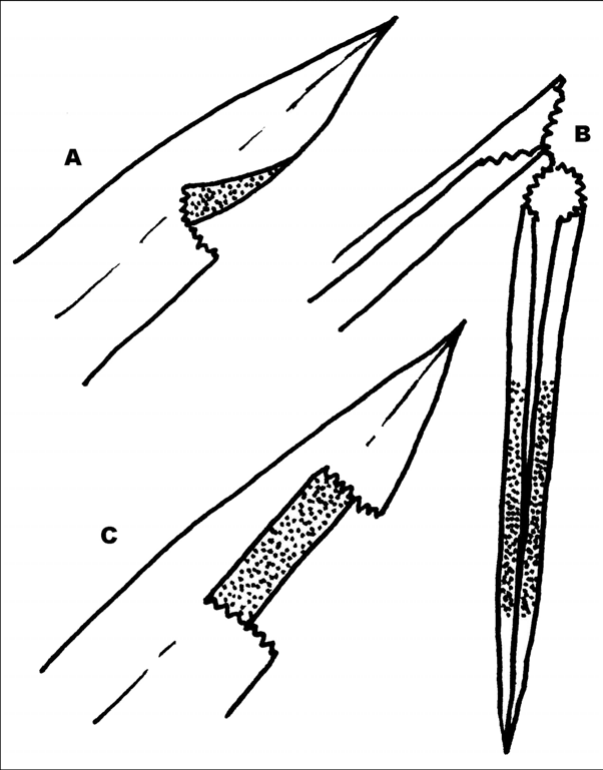
Larval shelters of *Vettius coryna coryna* at YBS. a) Shelter constructed by 1^st^ instar; b) shelter constructed 4^th^/5^th^ instar; c) Shelter constructed by 3^rd^ instar.

**Figure 4.**
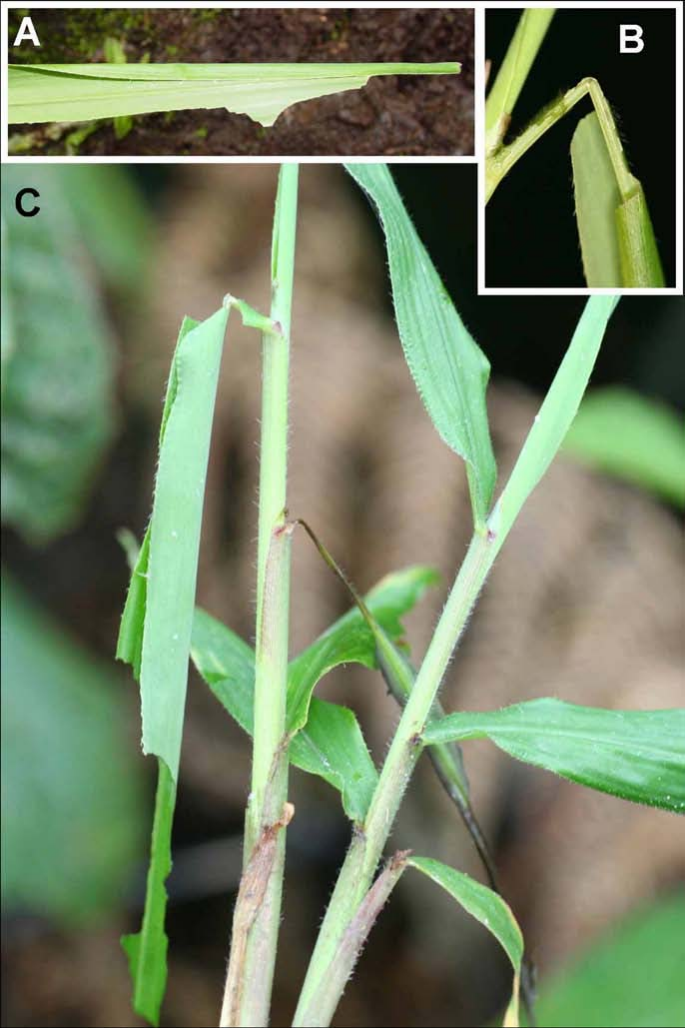
Larval shelters of *Vettius coryna coryna* at YBS. a) Shelter built by 3^rd^ instar; b) detail of positioning cuts at base of foodplant leaf; c) in situ 5^th^ instar shelter.

Terminology for shelter types follows Greeney and Jones (2003). *First instar* (n = 27): larvae construct a Group I, type two, no-cut fold shelter by curling one edge of the leaf margin onto the dorsal surface of the leaf. Feeding begins immediately on the shelter lid, which allows it to be silked more tightly to the surface of the leaf, giving it the appearance of having been formed by a single cut into the leaf margin (Group II, type six, one-cut fold shelter; Figure 3a). Immediately after construction, the shelter is tubular in cross section and thinly dome-shaped when viewed from above. Once feeding begins, the shelter becomes a flattened pocket, appearing as an elongate triangle when viewed from above (Figure 3a). All first instar shelters were located at the apices of leaf blades. *Second instar* (n = 26): larvae continue to utilize their initial shelters. *Third instar* (n = 29): larvae were also found in no-cut fold shelters, but the shelters were generally much larger than those constructed during earlier instars. As described for first instar shelters, third instar shelters begin as a simple curl onto the dorsal surface of the leaf, and after feeding begins, take on an elongate triangular to roughly rectangular shape (Figures 3c, 4a). For both the first and second shelter constructed, feeding damage surrounding the shelter quickly obscures its original form. *Fourth instar* (n = 37): larvae were all found in Group III, type eight, two-cut fold shelters. Two major cuts were made from opposite sides of the leaf, nearly meeting at the midvein. Opposing leaf margins were drawn together with silk to form a shallow pocket, then a tiny positioning cut was made near the base of the leaf midvein, causing it to weaken and hang in a vertical position (Figures 3b, 4b, 4c). Little or no silk was found around these positioning cuts. Major cuts and the positioning cut were made in the leafs basal third. *Fifth instar* (n = 31): shelter construction was similar to that described for the fourth instar.

However, some curled with age, giving the shelter a tubular (rather than flattened) overall shape. Among fifth instar larvae continuously monitored in the field (n = 27), 21 built new shelters immediately prior to or immediately after molting. The remaining 6 constructed a fourth shelter. *Pupal shelter:* Only two pupae were encountered in the field. One was located in a shelter away from the foodplant, constructed with a living leaf of *Chusquea* Kunth (Poaceae). The other was found in a shelter made from an undamaged leaf of the foodplant. These shelters were Group I, type 2, no-cut fold shelters. Opposing leaf margins were drawn downward and slightly together, forming an inverted, open, canoe-shaped tent. The pupa was attached upside down to the ventral side of the leaf. Several heavy ties of sealing silk were located above and below the pupa in addition to many small ties crossing the midvein along the entire length of the shelter. A silk girdle crossed the thorax, and the cremaster was attached to one of the small crossties along the mid-vein.

### Adult behavior

Adults of *Vettius c. coryna* (Figure 2b) are common throughout the year at YBS. They are most frequently found along road cuts and in large clearings within the forest. Both sexes visit a variety of flowers, including species of *Heppiella* Regel. (Gesneriaceae), *Prunella* L. (Lamiaceae), *Verbena* L. (Verbenaceae), *Fuchsia* L. (Onagraceae), *Baccharis* L., *Munnozia* Ruiz & Pav., *Erato* DC., and *Adenostemma* J. R. Forst. & G. Forst. (Asteraceae). During overcast days and in early morning, adults are often seen basking on the upper surfaces of leaves with their hindwings spread and their forewings mostly closed. Males remain on perches throughout the day, usually no higher than two meters above the ground. They fly out to inspect butterflies of all sizes, including individuals many times larger than themselves. Females search for oviposition sites during periods of full sun from 10:00 to 14:00 hrs, touching down briefly on the upper surfaces of narrow-bladed grasses, and pausing occasionally to bask. A single oviposition event was witnessed on 31 July 2001, at 11:30 hrs. After basking for several minutes, the female flew to the base of a nearby clump of grasses and curled her abdomen under a young leaf-blade to lay a single egg on its undersurface. She then flew rapidly out of the area.

## Discussion

Larval foodplants have been reported for the following *Vettius* species: *V. fantasos* (Cramer, 1780), *V. tertianus* (Herrich-Schäffer, 1869), *V. lucretius* (Latreille, [1824]), *V. diversa* (Herrich-Schäffer, 1869), *V. artona* (Hewitson, 1868), *V. diana* (Plötz, 1886), *V. marcus* (Fabricius, 1787), *V. lafrenaye* (Latreille, 1824), and *V. aurelius* (Plötz, 1882). For *V. fantasos*, Sepp (1847) noted *Panicum ramosum* L. (Poaceae), as a larval foodplant, as repeated by Hayward (1947). Draudt (1924) noted that the “green larvae live on grasses such as *Panicum ramosum*.” Later, Kendall (1976) reported *V. fantasos* larvae from Tamaulipas, Mexico, feeding on “*Lasiacis sp. (? ruscifolia)*,” but changed that determination in Kendall and McGuire (1984) to “*Lasiacis* sp., probably *divaricata* (L.)” (Poaceae). Kendall and McGuire (1984) speculate that *Arnudo donax* L. or *Phragmites australis* (Cav.) Trin. ex Steud. (both Poaceae) may serve as suitable hosts in south Texas. Recently, Janzen and Hallwachs (2007) reported *Lasiacis sorghoidea* (Desv. ex Hamilton) A.S. Hitch. & Chase, and *Lasiacis procerrima* (Hack.) A.S. Hitch. & Chase (both Poaceae) as popular larval foodplants for *V. fantasos* in Guanacaste Costa Rica, but also recorded *Lasiacis ruscifolia* (Kunth) A.S. Hitch. & Chase, two undetermined species of *Lasiacis* (Griseb.) A.S. Hitch., *Olyra latifolia* L., *Panicum trichidiachne* Doell, and *Panicum trichoides* Sw. (all Poaceae) as additional larval foodplants used by *V. fantasos* in the region.

Moss (1949) was the first to report a larval foodplant for *V. tertianus* (repeated by Silva et al. 1968). He reared one adult from a species of *Scleria* Berg. (Cyperaceae), and briefly described the pupa as being “brown and rounded at the extremities and is tightly packed in a closed web.” Orivel and Dejean (2000) described and illustrated the life cycle of *V. tertianus*, found on *Aechmea mertensii* (G. Mey.) Schult. (Bromeliaceae) in French Guiana. A brief description of the egg and late instar was given, along with photos of the egg, second and last instars, and pupa. The authors also investigated a possible association between immatures of *V. tertianus* and various species of ants. Janzen and Hallwachs (2007) reported another bromeliad (Bromeliaceae), *Catopsis nutans* (Sw.) Griseb., as a larval foodplant for *V. tertianus* in Guanacaste, Costa Rica.

Moss (1949) provided a brief description of the larva of *V. lucretius*, which he regularly found at the fruit market at Para, Brazil, living “amongst the sharp-spined leaves which grow at the top of the pineapple fruits” (Bromeliaceae). Moss' entire description of the larva of *V. lucretius* and its habits was reproduced by Brown and Heineman(1972).

Reported larval foodplants for other species of *Vettius* include “palmeiras” (Arecaceae) for *V. diversa* (K. Brown 1992), *Bromelia* L. (Bromeliaceae) for *V. artona* (Zikán and Zikán 1968) and *V. diana diana* (Zikán and Zikán 1968, repeated by Silva et al. 1968), “palmeira marajá,” or *Bactris* Jacq. ex Scop. (Arecaceae), for *V. marcus* (Silva et al. 1968), and “bambú añao e tawuara” (Silva et al. 1968) or “‘taquara’ (*Guadua*? sp.)” (Zikán and Zikán 1968) (Poaceae) for *V. lajrenaye lafrenaye*. In addition, Janzen and Hallwachs (2007) reported larval foodplants for *Vettius lafrenaye pica* (Herrich-Schäffer, 1869), including *Olyra latifolia* and an unidentified grass (both Poceae); *Vettius diversa maeon* (Mabille, 1891), including *Lasiacis ruscifolia*, an undetermined *Lasiacis* species, and another undetermined grass (all Poaceae); *Vettius coryna conka* Evans, 1955, including *Lasiacis nigra* Davidse, *Lasiacis rhizophora* (E. Fourn.) A.S. Hitch. & Chase, *Lasiacis ruscifolia*, and an undetermined grass species; and *Vettius aurelius* (Plötz, 1882), including five species of *Lasiacis, Oplismenus hirtellus* (L.) P. Beauv., *Panicum trichidiachne, Pharus parvifolius* Nash, *Urochloa anecta* (Hack, ex T. Dur. & Schinz) O. Morrone & F. Zuloaga, and two undetermined species of grass.

Larvae of many hesperiine skippers are polyphagous, yet tend to feed on monocots with similar structural characteristics (Scott 1992). Plant architecture may be a constraint for foodplant suitability to skippers such as *V. c. coryna* that build elaborate larval shelters. Indeed, the two grass species upon which we have found *V. c. coryna* larvae are similar in that both are relatively long-bladed with a pronounced petiole, and both are slightly to moderately pubescent. However, *Paspalum* sp. is a low-growing grass, rarely taller than 60cm, with blades roughly 15–20 cm in length. *Pennisetum tristachyon*, on the other hand, is often over two meters tall, with blades roughly 30–70 cm in length. Both grasses are found exclusively in areas of forest disturbance. We would not be surprised to discover that *V. c. coryna* utilizes different grasses as larval foodplants in other parts of its range. In western Ecuador (Pichincha Province), we have encountered *V. c. coryna* where neither *Paspalum* nor *Pennisetum* occur, although other (undetermined) long-bladed, moderately pubescent grasses were present.

*Vettius coryna coryna* is one of many tropical skippers whose life history was previously undescribed, despite the abundance of adults and immatures in disturbed areas. Life history data can be extremely valuable for testing phylogenetic hypotheses, when adequate comparative information is available (*e.g*., Judd 1998). For *Vettius*, a genus containing no fewer than 22 species, much additional work remains before we can assemble enough character information to place the observations presented here into a phylogenetic or revisionary context. It is our hope that this study will encourage similar observations on the life histories of common but poorly known tropical skippers.

### Editor's note

Paper copies of this article will be deposited in the following libraries. Senckenberg Library, Frankfurt Germany; National Museum of Natural History, Paris, France; Field Museum of Natural History, Chicago, Illinois USA; the University of Wisconsin, Madison, USA; the University of Arizona, Tucson, Arizona USA; Smithsonian Institution Libraries, Washington D.C. USA; The Linnean Society, London, England.
